# Early sitting within 72 hours in the intensive care unit and post–intensive care outcomes: a multicenter cohort study

**DOI:** 10.1016/j.aicoj.2026.100181

**Published:** 2026-10-07

**Authors:** Shinichi Watanabe, Yuki Iida, Yorihide Yanagita, Ayato Shinohara, Tomoyuki Morisawa, Ryo Kozu, Yusuke Kawai, Junji Hatakeyama, Shigeaki Inoue, Osamu Nishida

**Affiliations:** a Department of Physical Therapy, Faculty of Rehabilitation, Gifu University of Health Science, 2-92 Higashiuzura, Gifu, 500-8281, Japan; b Department of Physical Therapy, School of Health Sciences, Aichi Shukutoku University, 2-9 Katahira, Nagakute, 480-1197 Japan; c Department of Physical Therapy Science, Nagasaki University Graduate School of Biomedical Sciences, 1-7-1 Sakamoto, Nagasaki, Nagasaki, 852-8520, Japan; d Department of Rehabilitation, Fujita Health University Hospital, 1-98 Dengakugakubo, Kutsukake-Cho, Toyoake, Aichi 470-1192 Japan; e Department of Rehabilitation, Kobe Rehabilitation Hospital, 5-1 Kanocho, Chuo-ku, Kobe, Hyogo, 650-8570 Japan; f Department of Nursing, Fujita Health University Hospital, 1-98 Dengakugakubo, Kutsukake-Cho, Toyoake, Aichi 470-1192 Japan; g Advanced Critical Care and Emergency Center, Yokohama City University Medical Center, 4-57 Urafune-cho, Minami-ku, Yokohama, 232-0024, Japan; h Department of Emergency and Critical Care Medicine, Wakayama Medical University, 811-1 Kimiidera, Wakayama 641-8509 Japan; i Department of Anesthesiology and Critical Care Medicine, School of Medicine, Fujita Health University, 1-98 Dengakugakubo, Kutsukake-Cho, Toyoake, Aichi 470-1192 Japan

**Keywords:** Early mobilization, Intensive care unit, Sitting, Cognitive impairment, Post–intensive care syndrome

## Abstract

**Background and purpose:**

Early mobilization is recommended in the intensive care unit (ICU), yet clinically meaningful early milestones remain unclear. We investigated whether achievement of sitting within 72 h of ICU admission was associated with post–intensive care outcomes among ICU survivors who underwent rehabilitation.

**Methods:**

In this multicenter observational cohort study, we included mechanically ventilated adult ICU survivors who underwent rehabilitation. Early sitting was defined as achievement of sitting at the bedside within 72 h after ICU admission. The primary outcome was death or functional decline at 3 months after hospital discharge, assessed using the Kihon Checklist. Prespecified secondary and exploratory outcomes included domain-specific impairments (physical, cognitive, and depressive), hospital readmission, and a composite of readmission or death within 3 months. Overlap propensity score weighting was used to balance baseline covariates. Multiple prespecified sensitivity analyses (48-h alternative definition, landmark analysis, exclusion of never-sitters, adjustment for Day-3 clinical status and rehabilitation dose) and Benjamini–Hochberg (BH) multiplicity adjustment were performed.

**Results:**

Among 423 patients, 173 achieved early sitting (≤72 h) and 250 did not. After overlap weighting, baseline characteristics were balanced. Early sitting was not associated with the primary outcome (adjusted risk ratio [aRR] 0.94, 95% confidence interval [CI] 0.80–1.09; p = 0.401). In exploratory analyses, early sitting was associated with a lower risk of cognitive domain impairment or death (aRR 0.80, 95% CI 0.67–0.97; p = 0.024; BH-adjusted p = 0.096) and of readmission or death (aRR 0.53, 95% CI 0.32–0.88; p = 0.014; BH-adjusted p = 0.027). No individual KCL item showed a clear association after weighting, and the cognitive association was not observed under a stricter definition. Associations with readmission-related outcomes were attenuated after adjustment for Day-3 clinical status.

**Conclusion:**

Achieving sitting within 72 h of ICU admission was not associated with death or functional decline at 3 months. The exploratory associations with cognitive-functional impairment and readmission-related outcomes may reflect the selection of clinically stable patients for early sitting rather than an effect of sitting itself. These findings support the feasibility of early sitting in clinically stable patients but do not establish early sitting as a therapeutic target; randomized trials are needed.

## Highlights

• Early sitting was not associated with 3-month death or functional decline
• Exploratory associations with cognitive-functional and readmission outcomes
• The cognitive association was not robust to a stricter KCL definition
• Associations may reflect selection of clinically stable patients for sitting
• Early sitting is feasible in stable patients; randomized trials are needed

## Introduction

Advances in intensive care medicine have substantially improved survival of critically ill patients. Consequently, increasing attention has shifted toward long-term morbidity among intensive care unit (ICU) survivors, commonly referred to as post–intensive care syndrome (PICS), which encompasses persistent physical, cognitive, and psychological impairments after critical illness [[1], [2], [3]]. These sequelae are associated with reduced functional independence, impaired quality of life, and increased healthcare utilization following hospital discharge [4,5]. Accordingly, prevention of long-term disability has become a major priority in contemporary critical care practice [1].

Early mobilization during ICU stay has been recommended as a key strategy to mitigate complications related to immobility and critical illness. Randomized and observational studies have demonstrated that early rehabilitation is feasible and may improve functional recovery and other patient outcomes [[6], [7], [8]]. As a result, international clinical practice guidelines recommend initiation of mobilization as early as clinically feasible [7]. However, implementation remains heterogeneous across ICUs, and clinically meaningful milestones and optimal timing of mobilization remain incompletely defined [9,10].

Most prior studies have focused on rehabilitation intensity or cumulative mobilization dose, as well as achievement of advanced mobility milestones such as standing or ambulation [11,12]. More recently, approaches that better capture heterogeneity in patient response and optimize mobilization "dose" at the individual level have gathered increasing interest [[12], [13], [14]]. In this context, early intermediate mobility milestones may be pragmatic and clinically meaningful goals achievable in a broad ICU population. Sitting at the edge of the bed is one of the earliest active mobilization goals and reflects recovery of postural control, cardiopulmonary tolerance to upright positioning, and neurological responsiveness. Recent evidence further suggests that upright sitting may improve oxygenation compared with semi-recumbent positioning in spontaneously breathing ICU patients receiving respiratory support [15], providing additional physiological rationale for early achievement of this mobility milestone. Previous studies have suggested that initiation of mobilization within the first 48–72 h may be associated with improved outcomes in critically ill patients [[16], [17], [18]]. However, whether achieving sitting within a clinically relevant early time window, such as within the first 72 h after ICU admission, is associated with improved post-ICU outcomes remains to be elucidated. In the present study, we selected 72 h as the primary threshold because it represents a clinically pragmatic early window during which sitting can be attempted in a broad population of mechanically ventilated ICU patients.

Therefore, in this multicenter cohort study using propensity score overlap weighting, we investigated the association between early achievement of sitting within 72 h of ICU admission and post–intensive care outcomes among patients who survived ICU hospitalization. In addition, we explored dose–response relationships between time to first sitting and outcomes using restricted cubic spline analyses and assessed robustness through multiple prespecified sensitivity analyses.

## Methods

### Study design and setting

This study is a secondary analysis of data derived from a multicenter, prospective, observational cohort study conducted as part of the Japanese Research of Rehabilitation and Risk Factors on Post-Intensive Care Syndrome (J-RELIFE) project (UMIN000036503) conducted across 24 ICUs in Japan (Supplementary Figure S1, Supplementary Table S1). The study was designed to investigate PICS, including physical, cognitive, and psychological impairments, and their associated risk factors in critically ill patients discharged from ICUs [19].

### Participants

The J-RELIFE registry consecutively enrolled adult patients admitted to participating mixed medical–surgical ICUs between October 2021 and December 2023. All patients were screened within 48 h of ICU admission by trained investigators and dedicated rehabilitation teams at each participating institution. Patients requiring invasive mechanical ventilation for ≥48 h who received rehabilitation during ICU hospitalization were enrolled as part of routine clinical care. In contrast, patients aged <20 years; those with preexisting severe motor impairment or central nervous system disorders preventing mobility assessment; those who had lost ambulatory independence prior to hospital admission; those with severe communication impairment precluding evaluation; those under coronavirus disease 2019 quarantine restrictions; those who declined participation; those who did not undergo rehabilitation intervention; or those receiving end-of-life care were excluded.

Temporary delirium at ICU admission was not considered an exclusion criterion, and enrollment proceeded once delirium improved or surrogate consent was obtained. Patients who died during ICU hospitalization were not included in post-ICU outcome analyses because follow-up outcomes could not be assessed.

### Exposure: time to first sitting achievement

The exposure of interest was time to first sitting, defined as the interval between ICU admission and successful achievement of sitting at the bedside or edge of bed with rehabilitation assistance, corresponding to active mobilization at or above the sitting level. Across participating centers, rehabilitation followed a standardized, stepwise mobilization framework comprising five activity levels: passive movement and respiratory therapy (level 1), active range-of-motion exercise (level 2), sitting at the bedside (level 3), standing (level 4), and ambulation (level 5) [8,16]. Trained physiotherapists assessed patients daily, initiated rehabilitation as early as clinically feasible, and selected the highest achievable level at each session according to the patient's physiological status, level of consciousness, and ability to cooperate. Mobilization at level 3 or higher was withheld when any of the physiological instability criteria listed in Supplementary Table S2 were present, and the decision to continue, temporarily suspend with subsequent reassessment, or resume mobilization was based on the patient's condition. Sitting therefore represented the first level of active, antigravity mobilization; after first sitting, patients could progress to standing and ambulation in subsequent sessions as tolerated, while lower-level exercises were continued as appropriate.

For the primary analysis, early sitting was defined as achievement of sitting within 72 h after ICU admission. Patients achieving sitting beyond 72 h or not achieving sitting during ICU stay were categorized into the delayed sitting group [[16], [17], [18]]. A prespecified sensitivity analysis applied an alternative threshold of ≤48 h. We selected 72 h a priori as the primary cutoff because it represents a pragmatic and clinically achievable early mobilization window across participating ICUs.

### Outcomes

Data, including baseline demographics, comorbidities, ICU treatments, rehabilitation parameters, and functional assessments, were prospectively collected from ICU admission to 3 months post-hospital discharge. The primary outcome was functional status at 3 months after hospital discharge, assessed using the Kihon Checklist (KCL) [[19], [20], [21]]. Global functional decline was defined as a total KCL score ≥8, according to established criteria. In addition, a composite outcome of death or functional decline by 3 months after hospital discharge was evaluated to account for mortality after ICU discharge. Only deaths confirmed during the 3-month follow-up period were included in the composite outcomes. Patients lost to follow-up without confirmed vital status were not classified as deceased and were handled as missing for the corresponding post-discharge outcome assessment. Domain-specific impairments based on individual KCL components, including physical function, cognitive function, and depressive mood, were also assessed. These domains represent key components of post–intensive care functional vulnerability and long-term disability among ICU survivors.

Secondary outcomes included functional decline among survivors and domain-specific impairment combined with death, including physical domain impairment or death, cognitive domain impairment or death, and depressive mood impairment or death. Healthcare utilization outcomes comprised hospital readmission within 3 months after discharge and a composite outcome of readmission or death within the same follow-up period. All-cause mortality by 3 months after hospital discharge was additionally evaluated.

Composite outcomes incorporating death were prespecified to account for competing risk related to mortality after ICU discharge.

### Covariates

Potential confounders were prespecified based on clinical relevance and prior evidence indicating associations with both the timing of mobilization and post–intensive care outcomes [[22], [23], [24]]. Baseline variables measured at ICU admission were included to account for differences in demographic characteristics, illness severity, premorbid vulnerability, treatment exposure, and institutional practice patterns.

Covariates included age, sex, body mass index, ICU admission category (respiratory, cardiovascular, gastrointestinal, sepsis excluding pneumonia, or other causes), Acute Physiology and Chronic Health Evaluation II (APACHE II) score, Clinical Frailty Scale [25], Charlson Comorbidity Index, use of blood purification therapy, sedation use during ICU stay, vasopressor use, and participating facility. For prespecified sensitivity analyses, we additionally used variables prospectively collected in the J-RELIFE registry, including Day 1–3 clinical status (RASS, continuous sedation, continuous vasopressor use, CAM-ICU delirium, and fulfillment of mobilization criteria) and rehabilitation dose (mean daily rehabilitation duration during the ICU stay and daily rehabilitation minutes on Days 1–3).

### Statistical analysis

Continuous variables are presented as mean ± standard deviation or median (interquartile range), as appropriate, whereas categorical variables are summarized as counts and percentages. Differences between patients who achieved sitting within 72 h after ICU admission (early sitting group) and those who did not (delayed sitting group) were adjusted using propensity score–based overlap weighting to account for baseline imbalances [26]. Propensity scores were estimated using multivariable logistic regression including clinically relevant baseline covariates—age, sex, body mass index, ICU admission category, APACHE II score, Clinical Frailty Scale and Charlson Comorbidity Index, blood purification therapy, sedation during ICU stay, vasopressor use, and participating facility. Overlap weighting was applied to generate weighted populations with balanced covariate distributions between groups. Covariate balance before and after weighting was assessed using standardized mean differences (SMDs), with an absolute SMD < 0.1 indicating adequate balance [27].

Associations between early sitting and clinical outcomes were evaluated using overlap-weighted generalized linear models with a Poisson distribution and log link to estimate adjusted risk ratios (aRRs) and corresponding 95% confidence intervals (CIs). Robust variance estimators were used to account for weighting-induced variance. To evaluate potential dose–response relationships, restricted cubic spline models with four knots placed at the 5th, 35th, 65th, and 95th percentiles of the time-to-first-sitting distribution were fitted to examine associations between time to first sitting and outcomes [28]. Models were adjusted for age, sex, body mass index, APACHE II score, Clinical Frailty Scale, and Charlson Comorbidity Index, with robust variance estimators. The reference value was set at 72 h after ICU admission. Overall and nonlinear associations were assessed using Wald χ^2^ tests.

Prespecified sensitivity analyses included a landmark analysis restricted to patients with an ICU length of stay (LOS) ≥72 h and an alternative exposure definition of early sitting as ≤48 h after ICU admission. Additional prespecified sensitivity analyses were conducted to address potential biases specific to the timing exposure: (a) exclusion of patients who did not achieve sitting during ICU stay (never sitters); (b) adjustment of the propensity score model for Day 3 clinical status (RASS, continuous sedation, continuous vasopressor use, CAM-ICU delirium, and fulfillment of mobilization criteria); (c) adjustment for refined illness severity (SOFA total score, sepsis, septic shock, and extracorporeal membrane oxygenation [ECMO]); and (d) a comprehensive model additionally adjusting for mean daily rehabilitation duration during the ICU stay together with (b) and (c). Results are shown in Supplementary Tables S8–S11 and Supplementary Figures S4–S5. All sensitivity analyses were conducted using the same overlap-weighting framework as the primary analysis. The effective sample size after overlap weighting was calculated as (∑w)^2^/∑w^2^ to evaluate information retention following weighting. E-values were calculated to assess the potential influence of unmeasured confounding [29].

To address multiplicity, we applied the Benjamini–Hochberg (BH) procedure to control the false discovery rate within prespecified outcome families: (1) the primary composite outcome (no adjustment, single hypothesis); (2) secondary domain-specific outcomes (n = 4); and (3) healthcare utilization and mortality outcomes (n = 3). Both unadjusted and BH-adjusted p-values are reported. Findings for secondary and exploratory outcomes were interpreted as hypothesis-generating (Supplementary Table S5).

Missing baseline covariate values were imputed with the mean. All statistical tests were two-sided, and a p value <0.05 was considered statistically significant. Statistical analyses were performed using R software (version 4.5.2; R Foundation for Statistical Computing, Vienna, Austria).

### Ethics statement

This multicenter observational cohort study was conducted across participating ICUs in Japan in accordance with the Declaration of Helsinki. The study protocol was approved by the Ethics Committee of Fujita Health University (HM21-077) and the institutional review boards of all participating institutions. Written informed consent was obtained from all participants or their legally authorized representatives prior to enrollment. In cases where patients were unable to provide consent at ICU admission, consent was obtained from surrogate decision-makers in accordance with local ethical regulations. This study is reported in accordance with the Strengthening the Reporting of Observational Studies in Epidemiology (STROBE) statement.

## Results

### Study population

A total of 2,606 adult patients who required mechanical ventilation for ≥48 h were screened. Of these, 2,183 patients were excluded because of pre-existing neurological or functional impairment, communication difficulties, absence of rehabilitation intervention, end-of-life care, death during ICU stay, or other predefined exclusion criteria. Finally, 423 patients were included in the analysis, comprising 173 patients in the early sitting group and 250 in the delayed sitting group (Fig. 1). Of the patients in the delayed sitting group, 231 (92.4%) eventually achieved sitting after 72 h, whereas 19 (7.6%) did not achieve sitting during ICU stay (never sitters). Baseline characteristics of the participants are presented in Table 1, with the three-group breakdown provided in Supplementary Table S3. Never sitters constituted a distinct subgroup with markedly greater illness severity, including higher SOFA total score, greater ECMO use, and longer ICU LOS and mechanical ventilation duration. Rehabilitation dose during ICU stay was higher in the early sitting group (mean daily rehabilitation minutes 29.2 ± 13.9 vs 20.0 ± 12.1, SMD 0.70; Table 1**)**. Before weighting, several differences in clinical characteristics were observed between the groups. After propensity score overlap weighting, covariate balance was substantially improved, with all SMDs below the prespecified threshold of 0.1, indicating adequate balance between the groups (Supplementary Table S4 and Supplementary Figure S2).

**Fig. 1:**
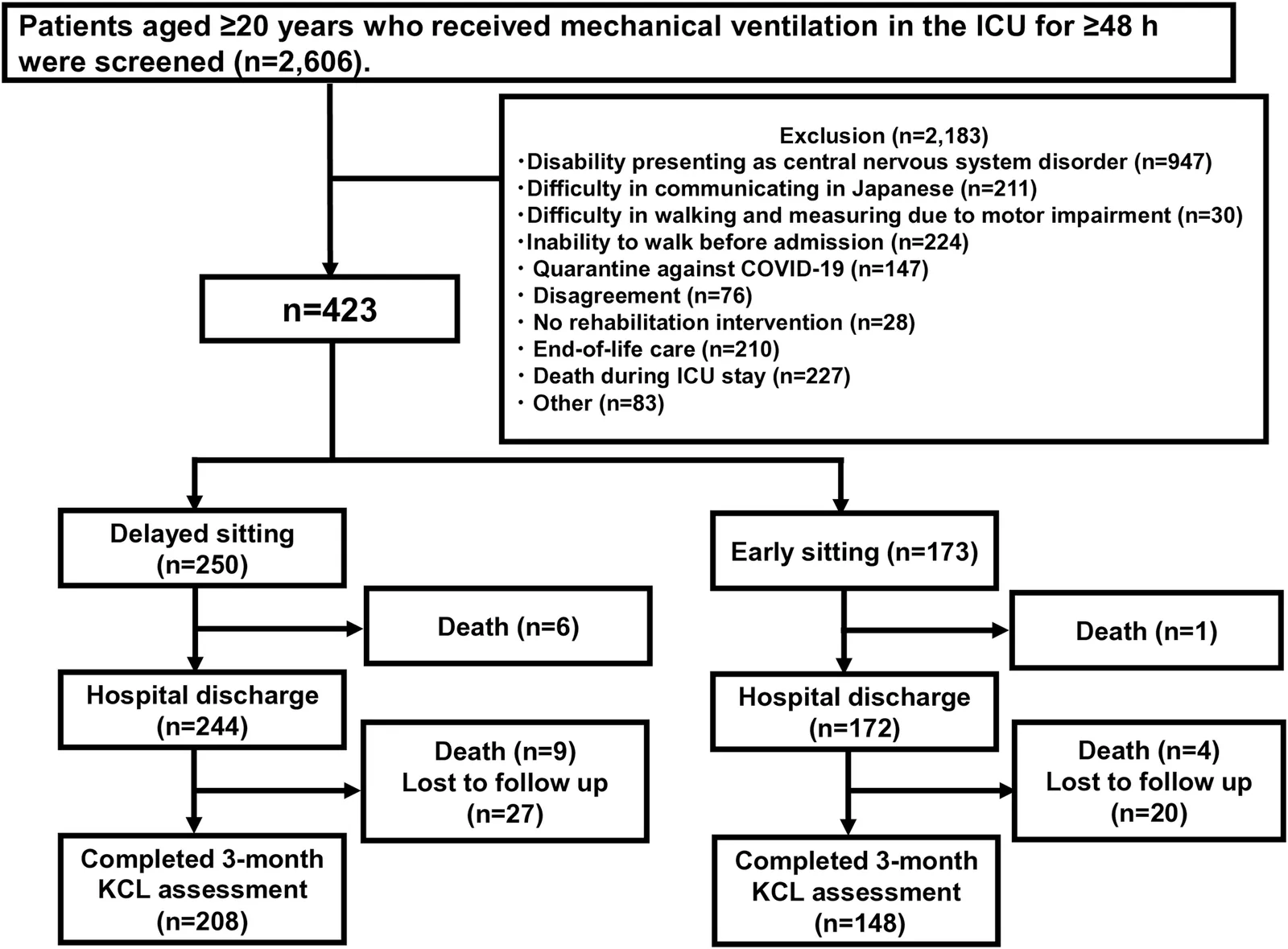
Flow diagram of patient selection and follow-up. Flow diagram of patient selection and inclusion in the study cohort. Patients admitted to participating intensive care units were screened according to the eligibility criteria, and the final analytical population was included in the outcome analyses. Vital status and readmission data were available for some patients who did not complete the 3-month KCL assessment; therefore, denominators differed across outcomes. Abbreviation: ICU, intensive care unit.

**Table 1:** Baseline characteristics of patients according to early versus delayed first sitting.

Variable	Delayed sitting (>72 h) (n = 250)	Early sitting (≤72 h) (n = 173)	SMD
Age, years	67.2 ± 13.4	68.4 ± 14.7	0.085
Male sex, n (%)	167 (66.8)	104 (60.1)	0.139
Body mass index, kg/m²	25.0 ± 5.2	24.3 ± 4.8	0.134
ICU admission category, n (%)			0.195
Respiratory	65 (26.0)	39 (22.5)	
Cardiovascular	94 (37.6)	62 (35.8)	
Gastrointestinal	32 (12.8)	29 (16.8)	
Sepsis (excluding pneumonia)	20 (8.0)	9 (5.2)	
Others	39 (15.6)	34 (19.7)	
APACHE II score	21.0 ± 8.1	20.6 ± 8.8	0.050
Clinical Frailty Scale	2.66 ± 1.13	2.81 ± 1.14	0.135
Charlson Comorbidity Index	1.78 ± 2.10	1.33 ± 1.67	0.237
Blood purification therapy, n (%)	57 (22.8)	33 (19.1)	0.092
Sedation use, n (%)	227 (90.8)	144 (83.7)	0.214
Vasopressor use, n (%)	192 (76.8)	138 (79.8)	0.072
Rehabilitation during ICU stay			
Day of rehabilitation initiation	2.0 ± 2.2	1.0 ± 0.7	0.591
Mean daily rehabilitation duration, min	20.0 ± 12.1	29.2 ± 13.9	0.704
Rehabilitation duration on ICU day 1, min	6.7 ± 10.1	13.8 ± 14.8	0.562
Rehabilitation duration on ICU day 2, min	11.1 ± 13.9	22.5 ± 16.8	0.736
Rehabilitation duration on ICU day 3, min	13.7 ± 14.7	30.2 ± 25.6	0.791

Values are presented as mean ± standard deviation or number (percentage), as appropriate. Standardized mean differences (SMDs) are shown to assess baseline imbalance between groups. An absolute SMD greater than 0.1 indicates meaningful imbalance between groups. ICU admission categories were defined based on the primary reason for ICU admission. Early sitting was defined as achievement of sitting within 72 h after ICU admission. Rehabilitation variables were measured after ICU admission and were not included in the propensity score model. Rehabilitation duration data were missing for one patient in the delayed sitting group (and, for ICU day 3, for one patient in the early sitting group).

Abbreviations: ICU, intensive care unit; APACHE II, Acute Physiology and Chronic Health Evaluation II; SMD, standardized mean difference.

### Association between early sitting and clinical outcomes

Overlap-weighted associations between early sitting and clinical outcomes are shown in Table 2 and Fig. 2. Early sitting within 72 h after ICU admission was not associated with the primary composite outcome of death or functional decline at 3 months after hospital discharge (aRR, 0.94; 95% CI, 0.80–1.09; p = 0.401).

**Table 2:** Overlap-weighted association between early sitting and clinical outcomes.

Outcome	Delayed sitting (>72 h) n/N (%)	Early sitting (≤72 h) n/N (%)	aRR (95% CI)	P value
Primary outcome				
Death or functional decline	173/223 (77.6%)	105/153 (68.6%)	0.94 (0.80–1.09)	0.401
Secondary outcomes				
Functional decline among survivors	158/208 (76.0%)	100/148 (67.6%)	0.94 (0.80–1.10)	0.446
Physical domain impairment or death	117/223 (52.5%)	72/153 (47.1%)	1.05 (0.81–1.36)	0.705
Cognitive domain impairment or death	166/223 (74.4%)	83/153 (54.2%)	**0.80 (0.67–0.97)**	**0.024**
Depressive mood impairment or death	114/223 (51.1%)	77/153 (50.3%)	0.96 (0.76–1.23)	0.769
Healthcare utilization				
Readmission within 3 months	40/220 (18.2%)	19/156 (12.2%)	**0.50 (0.29–0.89)**	**0.018**
Readmission or death	54/229 (23.6%)	23/158 (14.6%)	**0.53 (0.32–0.88)**	**0.014**
Mortality				
Death within 3 months	15/229 (6.6%)	5/158 (3.2%)	0.68 (0.20–2.31)	0.538

Associations between early sitting and clinical outcomes were evaluated using propensity score overlap weighting to adjust for baseline confounding. Adjusted risk ratios (aRRs) and 95% confidence intervals (CIs) were estimated using overlap-weighted Poisson regression models with robust (HC1) variance estimators with a log link and Poisson distribution. Event rates are presented as unweighted numbers and percentages. Each outcome was analyzed among patients with available data for that outcome; therefore, denominators differ across outcomes because vital status and readmission were ascertained for some patients who did not complete the 3-month Kihon Checklist assessment.

Abbreviations: aRR, adjusted risk ratio; CI, confidence interval; ICU, intensive care unit.

**Fig. 2:**
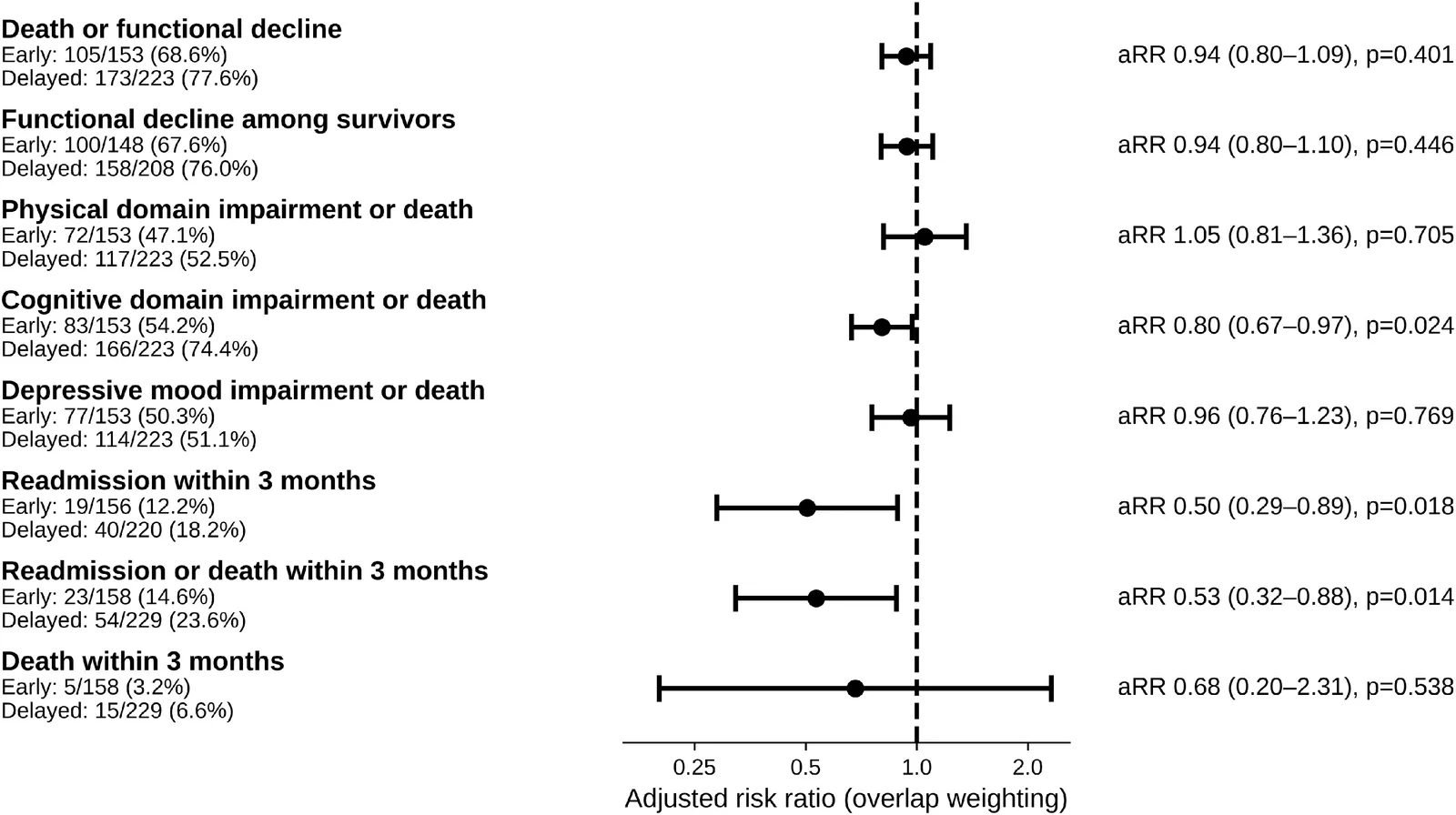
Association between early sitting and clinical outcomes using overlap weighting. Forest plot showing adjusted risk ratios for clinical outcomes between early sitting (≤72 h after ICU admission) and delayed sitting (>72 h). Estimates were derived from overlap-weighted Poisson regression models with robust variance estimators. Circles indicate point estimates, and horizontal lines represent 95% confidence intervals. The vertical dashed line indicates an aRR of 1.0. Event counts are presented as unweighted numbers (percentages). P values are unadjusted for multiple comparisons; Benjamini–Hochberg-adjusted p values are provided in Supplementary Table S5. Abbreviations: aRR, adjusted risk ratio; CI, confidence interval; ICU, intensive care unit.

Among the secondary and exploratory outcomes, early sitting was associated with a lower risk of cognitive domain impairment or death than delayed sitting (aRR, 0.80; 95% CI, 0.67–0.97; p = 0.024; BH-adjusted p = 0.096). Early sitting was also associated with a lower risk of hospital readmission (aRR, 0.50; 95% CI, 0.29–0.89; p = 0.018; BH-adjusted p = 0.027) and of the composite outcome of readmission or death (aRR, 0.53; 95% CI, 0.32–0.88; p = 0.014; BH-adjusted p = 0.027). No significant associations were observed for other outcomes, including physical domain impairment or death (aRR, 1.05; 95% CI, 0.81–1.36) and depressive mood impairment or death (aRR, 0.96; 95% CI, 0.76–1.23). Details of the multiple comparison adjustment are provided in Supplementary Table S5.

### Dose–response analysis

Restricted cubic spline analyses showed a continuous relationship between time to first sitting and outcomes (Fig. 3). For cognitive domain impairment or death, the overall association was statistically significant (χ² = 13.56, p = 0.004), with evidence of nonlinearity (χ² = 6.22, p = 0.045; Supplementary Table S6). For the composite of readmission or death, the overall association was not statistically significant (χ² = 4.27, p = 0.233).

**Fig. 3:**
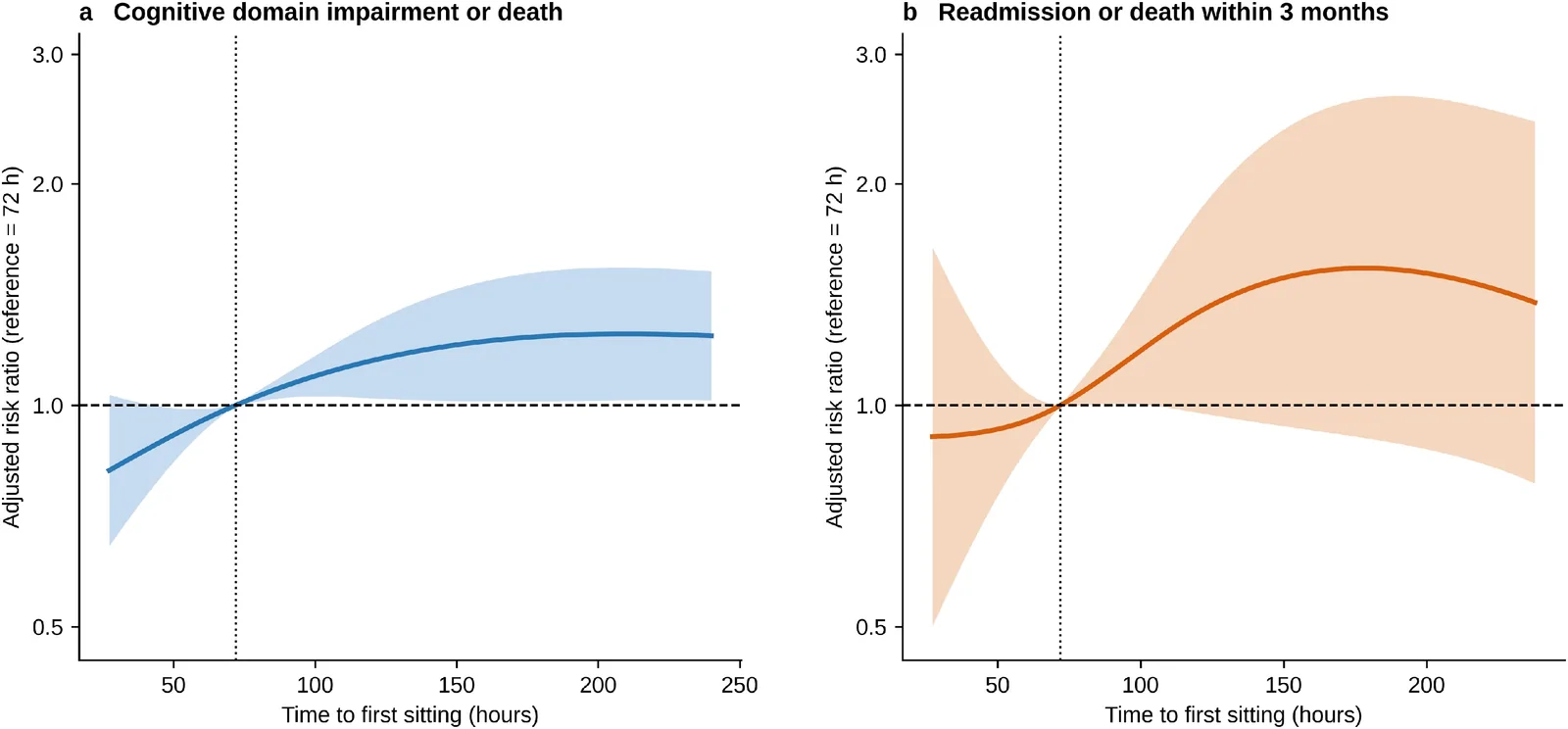
Restricted cubic spline analyses demonstrating the association between time to first sitting and clinical outcomes. Restricted cubic spline curves showing adjusted risk ratios for (a) cognitive domain impairment or death and (b) readmission or death within 3 months according to time to first sitting, with 72 h as the reference. Models used four knots at the 5th, 35th, 65th, and 95th percentiles and were adjusted for age, sex, body mass index, APACHE II score, Clinical Frailty Scale, and Charlson Comorbidity Index. Solid lines indicate point estimates, and shaded areas represent 95% confidence intervals. Curves are shown between the 10th and 90th percentiles of time to first sitting. The horizontal dashed line indicates an aRR of 1.0, and the vertical dotted line indicates 72 h. Abbreviations: ICU, intensive care unit; aRR, adjusted risk ratio; CI, confidence interval.

### Sensitivity analyses

Results of prespecified sensitivity analyses are presented in Supplementary Figure S3 and Supplementary Table S7 (original two-outcome sensitivity analyses) and are extended to all outcomes in Supplementary Tables S8–S11 and Supplementary Figures S4–S5. Across the primary analysis and four sensitivity scenarios—alternative exposure definition (≤48 h), landmark analysis (ICU LOS ≥ 3 days), exclusion of never sitters, and the combined ≤48-h definition with exclusion of never sitters—point estimates for cognitive domain impairment or death ranged from 0.77 to 0.82 (all raw p ≤ 0.05), whereas those for the composite outcome of readmission or death ranged from 0.50 to 0.55. Adjustment of the propensity score model for Day 3 clinical status (RASS, continuous sedation, continuous vasopressor use, CAM-ICU delirium, and fulfillment of mobilization criteria) attenuated the associations with readmission-related outcomes (RRs 0.56 and 0.60, both p > 0.15) but not with cognitive domain impairment or death (RR 0.76, p = 0.034). In the comprehensive model incorporating Day 3 clinical status, refined illness severity, and mean daily rehabilitation duration, point estimates remained directionally similar, although confidence intervals widened (Supplementary Table S10).

### Cognitive domain measurement

Because the KCL cognitive domain differs from standardized neuropsychological tests, we conducted an item-level analysis among survivors with available responses to each KCL item using the same overlap-weighted model as the primary analysis (Supplementary Table S12, Supplementary Figure S6). The unweighted proportions of positive responses for individual KCL items in the delayed vs early sitting groups were as follows: Q18 (memory) 20.7% vs 16.8% (RR 0.77, 95% CI 0.46–1.29); Q19 (telephone use, an instrumental activities of daily living [IADL] item) 56.2% vs 37.6% (RR 0.89, 95% CI 0.67–1.17); Q20 (orientation) 26.0% vs 27.7% (RR 0.97, 95% CI 0.65–1.43). No individual item showed a clear association after weighting. Using a more stringent alternative definition requiring ≥2 of the three items to be positive, the association was no longer observed (RR 1.07, 95% CI 0.67–1.70).

### Assessment of weighting performance

Propensity score distributions demonstrated adequate overlap between exposure groups after weighting (Supplementary Figure S7). Covariate balance improved substantially following overlap weighting, with all baseline covariates achieving absolute SMDs of <0.1 after weighting (Supplementary Table S4). Effective sample size was preserved after weighting, with an effective sample size of 298.8 in the primary analysis compared with the original sample size of 423, indicating stable estimation with minimal information loss (Supplementary Table S13).

### Sensitivity to unmeasured confounding

E-value analyses were conducted to assess the potential influence of unmeasured confounding (Supplementary Table S14). The E-value for the association between early sitting and cognitive domain impairment or death was 1.80 (95% CI limit, 1.21), and that for readmission or death was 3.15 (95% CI limit, 1.52), indicating that an unmeasured confounder would need to be associated with both early sitting and the outcome by a risk ratio of at least these magnitudes, beyond the measured covariates, to fully explain the observed associations.

## Discussion

In this multicenter observational cohort of ICU survivors, achieving sitting within 72 h was not associated with the primary composite outcome of death or functional decline at 3 months. Exploratory analyses suggested associations with a lower risk of cognitive-functional impairment and healthcare utilization. These signals were attenuated for readmission-related outcomes after adjustment for concurrent Day 3 clinical status, and the association with cognitive-functional impairment was not statistically significant after adjustment for multiple comparisons (BH-adjusted p = 0.096). Therefore, these findings should be interpreted as hypothesis-generating, and randomized trials are needed before causal inferences can be drawn.

We propose, as a hypothesis, that time to first sitting may serve as a mobilization milestone that reflects, and may facilitate, progression to more active mobilization. In our cohort, patients who achieved early sitting also received a greater rehabilitation dose during the first 3 ICU days (Table 1 and Supplementary Table S9), which is compatible with this hypothesis. In sensitivity analyses incorporating rehabilitation dose within the comprehensive adjustment model, the point estimates remained directionally similar, although confidence intervals widened. These analyses do not establish whether subsequent rehabilitation dose mediates the observed associations. Rather, the overall pattern is also compatible with the possibility that early sitting primarily identified patients who were clinically more stable or recovering more rapidly, in whom better outcomes might have occurred irrespective of mobilization. Our data cannot distinguish between these explanations.

Plausible biological mechanisms through which sustained early mobilization may influence long-term outcomes include mitigation of ICU-acquired weakness by reducing muscle wasting [30,31], preservation of neuromuscular function [32], and reduction of delirium and its downstream cognitive sequelae through earlier sensorimotor engagement and lighter sedation [2,33,34]. Recent evidence that upright sitting improves oxygenation compared with semi-recumbent positioning in spontaneously breathing ICU patients receiving respiratory support [15] provides additional physiological rationale. Nonetheless, the underlying mechanisms remain to be established, and our findings should be interpreted as hypothesis-generating.

Our findings complement prior results of randomized and observational studies demonstrating the benefits of structured early mobilization strategies in critically ill populations [6,34]. While earlier studies have primarily focused on mobilization intensity, cumulative rehabilitation dose, or achievement of advanced mobility milestones such as standing or ambulation, implementation research has demonstrated substantial heterogeneity in mobilization practices across ICUs worldwide [35,36]. Within this context, sitting may be better regarded as a feasible mobility milestone in appropriately selected, clinically stable patients than as a therapeutic target in itself. In light of the TEAM trial [11], which found no benefit and possible harm associated with higher-intensity mobilization, whether promoting sitting earlier—particularly before patients meet established stability criteria—would improve outcomes, and whether doing so would be safe, remains unknown.

From a recovery perspective, long-term functional trajectories following critical illness are increasingly recognized as dynamic processes influenced by early in-hospital exposures [23,37,38]. The present findings are consistent with the hypothesis that timing of initial upright activity is associated with post-ICU recovery trajectories, although causal inference from observational data remains limited. The application of propensity score overlap weighting achieved adequate covariate balance while preserving effective sample size, thereby improving comparability between the exposure groups [26,27]. In addition, E-value analyses were performed to quantify the potential influence of unmeasured confounding [29].

These findings may have implications for ICU rehabilitation practice. Although early mobilization is widely recommended, its implementation frequently varies because of hemodynamic instability, sedation exposure, and resource limitations. Sitting is a feasible early mobility milestone that can be achieved even when standing or ambulation is not yet possible. Our findings may be most useful for identifying clinically stable patients who are suitable for early sitting, using criteria such as those applied in this study (Supplementary Table S2). Whether targeting earlier sitting would improve outcomes requires evaluation in randomized trials; until then, our findings are consistent with the general recommendation to initiate mobilization as early as clinically feasible but do not establish a specific 72-h target as beneficial.

## Limitations

### Several limitations should be acknowledged

First, and most importantly, our exposure was inherently linked to early survival and clinical stability. Patients who died within 72 h, remained deeply sedated, or were hemodynamically unstable were unable to sit and were categorized in the delayed sitting group. Even after landmark analysis (restricted to patients surviving ≥3 days), sensitivity analyses excluding never sitters, and adjustment for Day 3 clinical status, we cannot fully exclude the possibility that the association between early sitting and outcomes reflects concurrent early recovery rather than a causal effect of sitting itself. However, Day 3 clinical status may itself represent an intermediate step on the causal pathway from early sitting to outcomes, and adjustment for such post-exposure variables can attenuate estimates of the total exposure effect through overadjustment. These two interpretations—residual confounding by indication and overadjustment for an intermediate variable—cannot be distinguished from observational data, and randomized trials are needed for definitive causal inference.

Second, the KCL cognitive domain is a brief three-item screening tool that includes an item assessing telephone use, which reflects IADL rather than pure neurocognitive function. Item-level analysis (Supplementary Table S12) showed no clear association for any individual item after weighting, and the association did not persist under a more stringent definition (≥2 of 3 items positive). Objective neuropsychological testing (e.g., Montreal Cognitive Assessment, Mini-Mental State Examination) was not performed. Therefore, our finding of cognitive impairment should be interpreted as cognitive-functional decline that includes IADL impairment rather than objective cognitive impairment. Moreover, the KCL has not been formally validated in ICU survivor populations.

Third, although we adjusted for mean daily rehabilitation duration, information on specific rehabilitation content (e.g., activity levels achieved, mobilization density, and involvement of physical therapists versus other staff) was not incorporated into the primary analysis. Time to first sitting should be interpreted as a mobility milestone rather than a standalone intervention.

Fourth, the final analytic cohort represented 16.2% of the initially screened mechanically ventilated population after excluding patients with early death, end-of-life care, pre-existing cognitive or communication impairment, or absence of rehabilitation intervention. Our findings therefore apply to ICU survivors who received rehabilitation and may not be generalizable to all mechanically ventilated ICU patients or to those with severe premorbid cognitive impairment.

Fifth, information regarding the specific types and dosages of sedative and analgesic agents was not available in this registry. Because sedation practices may directly influence both the feasibility and timing of mobilization as well as subsequent patient outcomes, residual confounding related to unmeasured pharmacological factors cannot be excluded. Sixth, heterogeneity in rehabilitation protocols, ICU organizational structures, and physical therapist staffing across centers may have influenced mobilization timing, resulting in a potential residual center-level confounding despite adjustment for participating facility. Seventh, outcome assessment relied on post-discharge follow-up, and unascertained post-discharge deaths among patients lost to follow-up could not be fully excluded, potentially leading to outcome misclassification.

## Conclusion

In this multicenter cohort study of ICU survivors who received rehabilitation, achieving sitting within 72 h of ICU admission was not associated with the primary composite outcome of death or functional decline at 3 months. Exploratory analyses showed associations with lower risks of cognitive-functional impairment and readmission-related outcomes; however, the cognitive association was not robust to a stricter definition of cognitive impairment, and the readmission-related associations were attenuated after adjustment for Day 3 clinical status. These associations may reflect the selection of clinically stable patients for early sitting rather than an effect of sitting itself. Our findings support the feasibility of early sitting in clinically stable patients but do not establish early sitting as a therapeutic target. Randomized trials with objective cognitive assessment are needed to determine whether promoting earlier sitting improves outcomes and is safe.

## Author contributions

Shinichi Watanabe and Yuki Iida conceived and designed the study. Shinichi Watanabe, Yuki Iida, Yorihide Yanagita, Ayato Shinohara, Tomoyuki Morisawa, Ryo Kozu, Yusuke Kawai, Junji Hatakeyama, Shigeaki Inoue, and Osamu Nishida contributed to data acquisition across participating centers. Shinichi Watanabe performed the statistical analyses and drafted the manuscript. All authors contributed to data interpretation, critically revised the manuscript for important intellectual content, and approved the final version of the manuscript.

## Consent to participate

Written informed consent was obtained from all participants in accordance with institutional and ethical guidelines.

## Consent for publication

Not applicable.

## Ethics approval

This study was approved by the institutional review boards of all participating institutions.

## Funding

This research did not receive any specific grant from funding agencies in the public, commercial, or not-for-profit sectors.

## Data availability

The datasets generated and analyzed during the current study are available from the corresponding author upon reasonable request, subject to institutional and ethical regulations.

## Declaration of competing interest

The authors declare that they have no competing interests related to this study.
