# Supplementary material for: Early sitting within 72 hours in the intensive care unit and post–intensive care outcomes: a multicenter cohort study
Source: Ann Intensive Care. 2026 Oct 7;16:100181. doi: 10.1016/j.aicoj.2026.100181 (PMC13681458; doi:10.1016/j.aicoj.2026.100181)
Supplement: Supplementary file 1 [file mmc1.docx]

(Supplementary File 1)

“Early Sitting Within 72 Hours in the Intensive Care Unit and Post–Intensive Care Outcomes: A Multicenter Cohort Study”

**Supplementary File – Figures**

Supplementary Figure S1: Map of participating sites.

Supplementary Figure S2: Covariate balance before and after overlap weighting.

Supplementary Figure S3. Graphical robustness summary across primary and sensitivity analyses.

Supplementary Figure S4: Forest plot: Primary analysis and four sensitivity analyses across all outcomes.

Supplementary Figure S5: Forest plot: Sensitivity analyses adjusting for time-varying confounders and rehabilitation dose.

Supplementary Figure S6: Forest plot: KCL cognitive domain item-level analysis.

Supplementary Figure S7: Propensity score distribution before and after overlap weighting.

**Supplementary File – Tables**

Supplementary Table S1: List of participating intensive care units and patient distribution.

Supplementary Table S2: Criteria for physiological instability.

Supplementary Table S3. Baseline characteristics of patients according to the three-group sitting classification.

Supplementary Table S4: Covariate balance before and after propensity score overlap weighting between early sitting and delayed sitting groups.

Supplementary Table S5. Multiple comparison adjustment (Benjamini–Hochberg and Bonferroni).

Supplementary Table S6: Tests for overall and nonlinear associations between time to first sitting and clinical outcomes using restricted cubic spline models.

Supplementary Table S7: Sensitivity analyses evaluating robustness of the association between early sitting and clinical outcomes.

Supplementary Table S8. Sensitivity analysis excluding never sitters.

Supplementary Table S9. Time-varying confounders and rehabilitation dose during ICU days 1–3, by exposure group.

Supplementary Table S10. Sensitivity analyses adjusting time-varying confounders, refined severity, and rehabilitation dose.

Supplementary Table S11. Extended sensitivity analyses across all outcomes and sensitivity analysis scenarios.

Supplementary Table S12. KCL cognitive domain: item-level analysis and alternative definitions.

Supplementary Table S13: Effective sample size after overlap weighting.

Supplementary Table S14: Sensitivity analysis for unmeasured confounding using E-values.

**Supplementary Figure S1.** **Map of participating sites.**

**Supplementary Figure S2. Covariate balance before and after overlap weighting.**

Absolute standardized mean differences (SMDs) for baseline covariates before (open circles) and after (filled circles) propensity score overlap weighting. SMDs before weighting correspond to those shown in Table 1. Facility indicators (24 centers) were also balanced after weighting (all absolute SMDs <0.001) and are not shown. The vertical dashed line indicates an absolute SMD of 0.10.

**Supplementary Figure S3. Graphical robustness summary across primary and sensitivity analyses.**

Adjusted risk ratios for cognitive domain impairment or death and readmission or death across the primary analysis (≤72 h), a landmark analysis restricted to patients with an ICU LOS ≥72 h, and an alternative exposure definition (≤48 h). Estimates were derived from overlap-weighted Poisson regression models with robust variance estimators. Circles indicate aRRs, and horizontal lines represent 95% CIs. The vertical dashed line indicates an aRR of 1.0.

Abbreviations: aRR, adjusted risk ratio; CI, confidence interval; ICU, intensive care unit; LOS, length of stay.

**Supplementary Figure S4. Forest plot: Primary analysis and four sensitivity analyses across all outcomes**

Forest plot showing overlap-weighted aRRs for all eight outcomes across five scenarios: primary (≤72 h), alternative definition (≤48 h), landmark analysis (ICU LOS ≥3 days), excluding never-sitters, and combined 48 h + excluding never-sitters. Point estimates were generally consistent across scenarios. Circles indicate point estimates, and horizontal lines indicate 95% CIs. Abbreviations: aRR, adjusted risk ratio; CI, confidence interval; ICU, intensive care unit; LOS, length of stay.

**Supplementary Figure S5. Forest plot: Sensitivity analyses adjusting for time-varying confounders and rehabilitation dose**

Forest plot showing effect of adding time-varying confounders (Day 3 status), refined severity (SOFA total, sepsis, septic shock, ECMO), and rehabilitation dose (mean daily rehab minutes) to the propensity score model. The point estimate for cognitive domain impairment or death was similar after all adjustments (comprehensive adjustment: RR 0.77, 95% CI 0.59–1.01), whereas associations with readmission-related outcomes were attenuated after adjustment for Day 3 status. Abbreviations: CI, confidence interval; ECMO, extracorporeal membrane oxygenation; PS, propensity score; RASS, Richmond Agitation–Sedation Scale; RR, risk ratio; SOFA, Sequential Organ Failure Assessment.

**Supplementary Figure S6. Forest plot: KCL cognitive domain item-level analysis.**

Forest plot showing overlap-weighted adjusted RRs for each KCL cognitive item (Q18: memory; Q19: telephone use; Q20: orientation) and for alternative composite definitions of cognitive impairment. The composite association (≥1 of 3 items, primary definition; RR 0.80, 95% CI 0.67–0.97) was not attributable to any single item, as no individual item showed a clear association. Under a more stringent definition (≥2 items positive), the association was not detectable (RR 1.07, 95% CI 0.67–1.70), suggesting that findings reflect cognitive-functional decline including IADL rather than objective cognitive impairment. Abbreviations: CI, confidence interval; IADL, instrumental activities of daily living; KCL, Kihon Checklist; RR, risk ratio.

**Supplementary Figure S7. Propensity score distribution before and after overlap weighting.**

Propensity score distributions for early and delayed sitting groups are shown before (Panel A) and after overlap weighting (Panel B). Overlap weighting improved covariate comparability by restricting inference to regions of common support, thereby satisfying the positivity assumption.

Abbreviations: PS, propensity score.

**Supplementary Table S1. List of participating intensive care units and patient distribution.**

| Characteristic | Participating ICUs (n=24) |
| --- | --- |
| Type of hospital, n (%) |  |
| Tertiary care hospital | 13 (54) |
| Secondary care hospital | 11 (46) |
| Type of ICU, n (%) |  |
| Mixed medical-surgical ICU | 24 (100) |
| System of ICU, n (%) |  |
| Closed ICU (intensivists have primary responsibility for patient management) | 5 (21) |
| Mandatory intensivists consultation for all ICU admissions | 13 (54) |
| Elective consultation (ICU physicians are primarily involved as consultants) | 6 (25) |
| Dedicated physiotherapist available, n (%) | 10 (42) |
| Staffing patterns on weekends, n (%) |  |
| Rehabilitation staff work only on weekdays | 4 (17) |
| Rehabilitation staff work on Saturdays and holidays | 12 (50) |
| Rehabilitation staff work every day | 8 (33) |

Data are presented as number (percentage).

ICU, intensive care unit.

**Supplementary Table S2. Criteria for physiological instability.**

| Physiological stability variable | Criterion |
| --- | --- |
| **Medical stability** |  |
| Bleeding tendency | If exercise may worsen the patient’s condition |
| Fever | >38.5 °C |
| Bed rest order | When the attending physician determines that the patient’s condition requires bed rest |
| **Cardiovascular stability** |  |
| Heart rate | <50 or >130 beats/min; decrease in heart rate or bradycardia after starting exercise |
| Mean arterial blood pressure | Mean arterial blood pressure <65 mmHg or >110 mmHg  Decrease of ≥20% in systolic or diastolic blood pressure |
| Vasoactive medication infusions^a^ | Increase in the dose of vasoactive medication within the previous 24 h |
| **Respiratory stability** |  |
| Fraction of inspired oxygen | >0.6 |
| Respiratory rate | >30 breaths/min |
| Pulse oximetry oxygen saturation | <90% |
| **Neurological stability** |  |
| Richmond Agitation–Sedation Scale | <−2 or >1 |
| Delirium | Glasgow Coma Scale motor response <M6 (unable to follow commands) |
| Glasgow Coma Scale | Motor response <M6 (unable to follow commands) |

The decision to discontinue the intervention, temporarily suspend it with subsequent reassessment, or resume it was based on the patient’s condition.

^a^noradrenaline, dopamine, adrenaline, vasopressin, milrinone, glyceryl trinitrate, sodium nitroprusside
**Supplementary Table S3**. **Baseline characteristics of patients according to the three-group sitting classification.**

| **Variable** | **Early (n=173)** | **Late (n=231)** | **Never (n=19)** | **P value** |
| --- | --- | --- | --- | --- |
| **Demographics** |  |  |  |  |
| Age (years) | 68.4±14.7 | 67.3±13.5 | 66.2±13.0 | 0.276 |
| Male sex | 104 (60.1%) | 152 (65.8%) | 15 (78.9%) | 0.192 |
| Body mass index (kg/m²) | 24.3±4.8 | 25.0±5.2 | 25.3±5.7 | 0.62 |
| **Illness severity** |  |  |  |  |
| APACHE II score | 20.6±8.8 | 21.0±8.1 | 20.5±8.3 | 0.863 |
| SOFA total score | 9.1±4.3 | 9.7±3.9 | 10.3±4.1 | 0.299 |
| SOFA respiratory | 1.8±1.1 | 2.2±1.2 | 2.2±1.3 | 0.018 |
| SOFA cardiovascular | 2.3±1.6 | 2.2±1.6 | 2.1±1.5 | 0.829 |
| SOFA neurological | 2.2±1.6 | 2.4±1.6 | 2.5±1.7 | 0.477 |
| **Comorbidity** |  |  |  |  |
| **Charlson Comorbidity Index** | 1.3±1.7 | 1.7±2.1 | 2.2±2.1 | 0.105 |
| Clinical Frailty Scale | 2.8±1.1 | 2.6±1.1 | 2.7±1.1 | 0.43 |
| Pre-admission Barthel Index | 97.6±8.1 | 98.3±6.1 | 99.7±1.1 | 0.317 |
| **ICU admission category** |  |  |  |  |
| Respiratory | 39 (22.5%) | 59 (25.5%) | 6 (31.6%) |  |
| Cardiovascular | 62 (35.8%) | 87 (37.7%) | 7 (36.8%) |  |
| Gastrointestinal | 29 (16.8%) | 32 (13.9%) | 0 (0.0%) |  |
| Sepsis (excluding pneumonia) | 9 (5.2%) | 20 (8.7%) | 0 (0.0%) |  |
| Others | 34 (19.7%) | 33 (14.3%) | 6 (31.6%) |  |
| **Clinical conditions** |  |  |  |  |
| Sepsis | 45 (26.0%) | 63 (27.3%) | 3 (15.8%) | 0.548 |
| Septic shock | 38 (22.0%) | 54 (23.4%) | 1 (5.3%) | 0.187 |
| ECMO | 1 (0.6%) | 21 (9.1%) | 4 (21.1%) | <0.001 |
| Blood purification therapy | 33 (19.1%) | 51 (22.1%) | 6 (31.6%) | 0.408 |
| Sedation use | 144 (83.7%) | 209 (90.5%) | 18 (94.7%) | 0.078 |
| Vasopressor use | 138 (79.8%) | 183 (79.2%) | 9 (47.4%) | 0.004 |
| **ICU course** |  |  |  |  |
| ICU length of stay (days) | 7.9±5.7 | 9.7±5.7 | 25.4±16.6 | <0.001 |
| Mechanical ventilation (days) | 5.4±7.9 | 7.1±7.3 | 28.4±24.9 | <0.001 |
| Hospital length of stay (days) | 43.0±33.0 | 47.4±34.0 | 81.2±39.3 | <0.001 |
| Mean daily ICU rehabilitation duration (min) | 29.2±13.9 | 20.4±11.8 | 16.1±14.8 | <0.001 |

Values are presented as mean ± standard deviation for continuous variables and n (%) for categorical variables. P values were derived using the Kruskal–Wallis test for continuous variables and the chi-square test for categorical variables. Early sitters were patients who achieved sitting within 72 h. Late sitters were patients who achieved sitting after 72 h. Never sitters were patients who did not achieve sitting during the ICU stay. Abbreviations: APACHE, Acute Physiology and Chronic Health Evaluation; ECMO, extracorporeal membrane oxygenation; ICU, intensive care unit; SOFA, Sequential Organ Failure Assessment.

**Supplementary Table S4. Covariate balance before and after propensity score overlap weighting between early sitting and delayed sitting groups.**

| **Covariate** | **Type** | **SMD Before Weighting** | **SMD After Weighting** |
| --- | --- | --- | --- |
| Age | Continuous | 0.085 | <0.001 |
| Male sex | Binary | 0.139 | <0.001 |
| Body mass index | Continuous | 0.134 | <0.001 |
| ICU admission category | Categorical | 0.195 | <0.001 |
| APACHE II score | Continuous | 0.050 | <0.001 |
| Clinical Frailty Scale | Continuous | 0.135 | <0.001 |
| Charlson Comorbidity Index | Continuous | 0.237 | <0.001 |
| Blood purification therapy | Binary | 0.092 | <0.001 |
| Sedation use | Binary | 0.214 | <0.001 |
| Vasopressor use | Binary | 0.072 | <0.001 |
| Facility (24 centers) | Binary indicators | ≤0.239 | <0.001 |

Standardized mean differences (SMDs) were calculated to assess balance in baseline covariates between patients who achieved sitting within 72 h after ICU admission (early sitting group) and those who did not (delayed sitting group). Covariate balance was evaluated before and after propensity score overlap weighting. An absolute SMD <0.1 was considered indicative of adequate balance. After overlap weighting, all baseline covariates achieved satisfactory balance between groups. SMDs are presented as absolute values; values before weighting are identical to those in Table 1.

Abbreviations: ICU, intensive care unit; APACHE II, Acute Physiology and Chronic Health Evaluation II; SMD, standardized mean difference.

**Supplementary Table S5**. **Multiple comparison adjustment (Benjamini–Hochberg and Bonferroni).**

| **Outcome** | **Category** | **Adjusted RR**  **(95% CI)** | **Raw p** | **BH adjusted p (all 8)** | **Bonferroni (all 8)** | **BH adjusted p (grouped)** | **Bonferroni (grouped)** |
| --- | --- | --- | --- | --- | --- | --- | --- |
| Death or functional decline | Primary | 0.94 (0.80–1.09) | 0.401 | 0.714 | 1 | 0.401 | 0.401 |
| Functional decline among survivors | Secondary | 0.94 (0.80–1.10) | 0.446 | 0.714 | 1 | 0.769 | 1 |
| Physical domain impairment or death | Secondary | 1.05 (0.81–1.36) | 0.705 | 0.769 | 1 | 0.769 | 1 |
| Cognitive domain impairment or death | Secondary | 0.80 (0.67–0.97) | 0.024 | 0.064 | 0.192 | 0.096 | 0.096 |
| Depressive mood impairment or death | Secondary | 0.96 (0.76–1.23) | 0.769 | 0.769 | 1 | 0.769 | 1 |
| Readmission | Healthcare util | 0.50 (0.29–0.89) | 0.018 | 0.064 | 0.144 | 0.027 | 0.054 |
| Readmission or death | Healthcare util | 0.53 (0.32–0.88) | 0.014 | 0.064 | 0.112 | 0.027 | 0.042 |
| Death within 3 months | Mortality | 0.68 (0.20–2.31) | 0.538 | 0.717 | 1 | 0.538 | 1 |

BH, Benjamini–Hochberg; CI, confidence interval; RR, risk ratio. “All 8” indicates adjustment to all eight outcomes. “Grouped” indicates adjustment within pre-specified outcome families: primary outcome (n=1, no adjustment); secondary domain-specific outcomes (n=4); and healthcare utilization and mortality outcomes (n=3). The primary outcome does not require multiplicity adjustment as it comprised a single hypothesis.

**Supplementary Table S6. Tests for overall and nonlinear associations between time to first sitting and clinical outcomes using restricted cubic spline models.**

| **Outcome** | **Overall association (χ²)** | **df** | **P value** | **Nonlinear association (χ²)** | **df** | **P value** |
| --- | --- | --- | --- | --- | --- | --- |
| Readmission or death within 3 months | 4.27 | 3 | 0.233 | 3.06 | 2 | 0.216 |
| Cognitive domain impairment or death | 13.56 | 3 | 0.004 | 6.22 | 2 | 0.045 |

Overall and nonlinear associations were evaluated using modified Poisson regression models (log link) incorporating restricted cubic splines with four knots, adjusted for age, sex, body mass index, APACHE II score, Clinical Frailty Scale, and Charlson Comorbidity Index, with robust variance estimators.

The overall association represents the joint Wald χ² test for the spline terms of time to first sitting, whereas the nonlinear association corresponds to the Wald χ² test for deviation from linearity. A two-sided p value <0.05 was considered statistically significant.

**Supplementary Table S7. Sensitivity analyses evaluating the robustness of the association between early sitting and clinical outcomes.**

| **Outcome** | **Primary analysis (≤72 h) RR (95% CI)** | **Landmark analysis (ICU LOS ≥72 h) RR (95% CI)** | **Alternative definition (≤48 h) RR (95% CI)** |
| --- | --- | --- | --- |
| Cognitive domain impairment or death | 0.80 (0.67–0.97) | 0.80 (0.66–0.97) | 0.77 (0.60–0.98) |
| Readmission or death within 3 months | 0.53 (0.32–0.88) | 0.55 (0.33–0.91) | 0.55 (0.30–1.01) |

Sensitivity analyses were conducted to assess the robustness of the primary findings. A landmark analysis restricted to patients who remained in the ICU for ≥72 h was performed to minimize exposure opportunity bias. Additionally, early sitting was alternatively defined as achievement of sitting within 48 h after ICU admission.

Propensity scores were estimated using baseline demographic and clinical variables, and overlap weighting was applied to achieve covariate balance between exposure groups. Adjusted risk ratios and 95% confidence intervals were estimated using weighted generalized linear models with a Poisson distribution and robust variance estimators.

Abbreviations: RR, risk ratio; CI, confidence interval; ICU, intensive care unit; LOS, length of stay.

**Supplementary Table S8**. **Sensitivity analysis excluding never sitters.**

| **Outcome** | **Primary Analysis (N=423), RR (95% CI)** | **Sensitivity Analysis: Excluding Never Sitters (N=404), RR (95% CI)** |
| --- | --- | --- |
| Death or functional decline | 0.94 (0.80–1.09) | 0.94 (0.81–1.10) |
| Functional decline among survivors | 0.94 (0.80–1.10) | 0.94 (0.80–1.11) |
| Physical domain impairment or death | 1.05 (0.81–1.36) | 1.04 (0.80–1.35) |
| Cognitive domain impairment or death | 0.80 (0.67–0.97) | 0.82 (0.68–0.99) |
| Depressive mood impairment or death | 0.96 (0.76–1.23) | 0.98 (0.76–1.25) |
| Readmission | 0.50 (0.29–0.89) | 0.46 (0.26–0.81) |
| Readmission or death | 0.53 (0.32–0.88) | 0.50 (0.30–0.82) |
| Death within 3 months | 0.68 (0.20–2.31) | 0.67 (0.20–2.27) |

The primary analysis (N=423) was compared with a sensitivity analysis excluding the 19 patients who did not achieve sitting during the ICU stay (N=404). Adjusted RRs and 95% CIs were estimated using overlap-weighted Poisson regression with robust variance estimators. The associations were consistent across the two analyses. Abbreviations: CI, confidence interval; ICU, intensive care unit; RR, risk ratio

**Supplementary Table S9**. **Time-varying confounders and rehabilitation dose during ICU days 1–3, by exposure group.**

| **Variable** | **Delayed sitting (n=250)** | **Early sitting (n=173)** | **SMD** | **P value** |
| --- | --- | --- | --- | --- |
| **Day 1 (ICU admission day)** |  |  |  |  |
| RASS | −3.09±1.80 | −2.32±1.76 | 0.432 | <0.001 |
| Continuous sedation | 227/248 (91.5%) | 144/172 (83.7%) | 0.239 | 0.020 |
| Continuous vasopressor | 184/248 (74.2%) | 134/172 (77.9%) | 0.087 | 0.419 |
| CAM-ICU positive (delirium) | 19/250 (7.6%) | 18/173 (10.4%) | 0.098 | 0.382 |
| Mobilization criteria met | 8/247 (3.2%) | 51/172 (29.7%) | 0.763 | <0.001 |
| SBT performed | 9/248 (3.6%) | 16/171 (9.4%) | 0.234 | 0.02 |
| Rehabilitation duration (min) | 6.73±10.07 | 13.83±14.77 | 0.562 | <0.001 |
| **Day 2** |  |  |  |  |
| RASS | −2.64±1.87 | −1.28±1.49 | 0.807 | <0.001 |
| Continuous sedation | 223/248 (89.9%) | 139/173 (80.3%) | 0.272 | 0.007 |
| Continuous vasopressor | 172/248 (69.4%) | 120/173 (69.4%) | <0.001 | 1.000 |
| CAM-ICU positive (delirium) | 22/250 (8.8%) | 33/173 (19.1%) | 0.300 | 0.003 |
| Mobilization criteria met | 19/247 (7.7%) | 112/173 (64.7%) | 1.475 | <0.001 |
| SBT performed | 22/248 (8.9%) | 45/173 (26.0%) | 0.464 | <0.001 |
| Rehabilitation duration (min) | 11.15±13.91 | 22.50±16.79 | 0.736 | <0.001 |
| **Day 3 (primary threshold)** |  |  |  |  |
| RASS | −2.34±1.86 | −0.71±1.13 | 1.055 | <0.001 |
| Continuous sedation | 206/249 (82.7%) | 112/172 (65.1%) | 0.410 | <0.001 |
| Continuous vasopressor | 152/249 (61.0%) | 89/172 (51.7%) | 0.188 | 0.071 |
| CAM-ICU positive (delirium) | 30/250 (12.0%) | 37/173 (21.4%) | 0.254 | 0.010 |
| Mobilization criteria met | 38/248 (15.3%) | 141/170 (82.9%) | 1.836 | <0.001 |
| SBT performed | 60/249 (24.1%) | 89/172 (51.7%) | 0.594 | <0.001 |
| Rehabilitation time (min) | 13.71±14.69 | 30.21±25.59 | 0.791 | <0.001 |
| **ICU rehabilitation dose summary** |  |  |  |  |
| Mean daily rehabilitation duration (min) | 20.05±12.11 | 29.22±13.88 | 0.704 | <0.001 |
| Total ICU rehabilitation duration (min) | 150.0 [90.0–245.0] | 178.0 [120.0–285.0] | 0.186 | 0.008 |
| Rehabilitation-related adverse event | 5/211 (2.4%) | 7/162 (4.3%) | 0.109 | 0.378 |

Values are shown as mean ± SD for continuous variables, n/N (%) for binary variables, or median [interquartile range] where indicated. SMD, standardized mean difference; RASS, Richmond Agitation–Sedation Scale; CAM-ICU, Confusion Assessment Method for the ICU; SBT, spontaneous breathing trial. P values were derived from Mann–Whitney U test (continuous) or Fisher's exact test (binary). Delirium was defined as a positive CAM-ICU result; “unassessable” responses were classified as negative.

**Supplementary Table S10**. **Sensitivity analyses adjusting for time-varying confounders, refined severity, and rehabilitation dose.**

| **Outcome** | **Primary RR (95% CI), p** | **+ Day 3 status** | **+ Refined severity** | **Comprehensive (all)** |
| --- | --- | --- | --- | --- |
| Death or functional decline | 0.94 (0.80–1.09), p=0.401 | 1.02 (0.82–1.28), p=0.840 | 0.95 (0.81–1.12), p=0.550 | 1.04 (0.83–1.31), p=0.713 |
| Cognitive domain impairment or death | 0.80 (0.67–0.97), p=0.024 | 0.76 (0.59–0.98), p=0.034 | 0.80 (0.65–0.97), p=0.027 | 0.77 (0.59–1.01), p=0.056 |
| Readmission | 0.50 (0.29–0.89), p=0.018 | 0.56 (0.24–1.26), p=0.161 | 0.53 (0.30–0.96), p=0.037 | 0.53 (0.22–1.25), p=0.146 |
| Readmission or death | 0.53 (0.32–0.88), p=0.014 | 0.60 (0.29–1.23), p=0.162 | 0.56 (0.33–0.93), p=0.026 | 0.58 (0.27–1.23), p=0.154 |
| Physical domain impairment or death | 1.05 (0.81–1.36), p=0.705 | 1.07 (0.74–1.56), p=0.724 | 1.05 (0.80–1.37), p=0.735 | 1.10 (0.75–1.61), p=0.625 |
| Depressive mood impairment or death | 0.96 (0.76–1.23), p=0.769 | 0.95 (0.67–1.35), p=0.782 | 0.99 (0.77–1.27), p=0.926 | 0.99 (0.69–1.42), p=0.968 |

Sensitivity analyses were performed by adding covariates to the propensity score model. Day 3 status included RASS, continuous sedation, continuous vasopressor use, positive CAM-ICU result, and fulfillment of mobilization criteria on Day 3. Refined severity included SOFA total score, sepsis, septic shock, and ECMO. The comprehensive model included all of the above covariates plus mean daily rehabilitation duration. The effective sample size decreased from 298.8 in the primary analysis to 149 in the comprehensive analysis because of the increased extremeness of the propensity scores. Abbreviations: CAM-ICU, Confusion Assessment Method for the Intensive Care Unit; CI, confidence interval; ECMO, extracorporeal membrane oxygenation; RASS, Richmond Agitation–Sedation Scale; RR, risk ratio; SOFA, Sequential Organ Failure Assessment.

**Supplementary Table S11**. **Extended sensitivity analyses across all outcomes and sensitivity analysis scenarios.**

| **Outcome** | **Primary (≤72h, N=423)** | **Alt definition (≤48h, N=423)** | **Landmark (ICU LOS ≥3d)** | **Excl. never-sitters (N=404)** | **≤48h + excl. never (N=404)** |
| --- | --- | --- | --- | --- | --- |
| Death or functional decline | 0.94 (0.80–1.09) p=0.401 | 0.95 (0.80–1.14) p=0.606 | 0.94 (0.80–1.10) p=0.435 | 0.94 (0.81–1.10) p=0.432 | 0.96 (0.80–1.14) p=0.626 |
| Cognitive domain impairment or death | 0.80 (0.67–0.97) p=0.024 | 0.77 (0.60–0.98) p=0.033 | 0.80 (0.66–0.97) p=0.026 | 0.82 (0.68–0.99) p=0.040 | 0.78 (0.61–1.00) p=0.046 |
| Readmission | 0.50 (0.29–0.89) p=0.018 | 0.50 (0.25–1.01) p=0.052 | 0.51 (0.29–0.89) p=0.019 | 0.46 (0.26–0.81) p=0.007 | 0.47 (0.24–0.94) p=0.032 |
| Readmission or death | 0.53 (0.32–0.88) p=0.014 | 0.55 (0.30–1.01) p=0.054 | 0.55 (0.33–0.91) p=0.019 | 0.50 (0.30–0.82) p=0.006 | 0.52 (0.28–0.95) p=0.034 |
| Physical domain impairment or death | 1.05 (0.81–1.36) p=0.705 | 1.07 (0.80–1.42) p=0.663 | 1.07 (0.82–1.39) p=0.609 | 1.04 (0.80–1.35) p=0.765 | 1.05 (0.79–1.40) p=0.717 |
| Depressive mood impairment or death | 0.96 (0.76–1.23) p=0.769 | 1.04 (0.80–1.34) p=0.781 | 0.97 (0.76–1.24) p=0.812 | 0.98 (0.76–1.25) p=0.865 | 1.05 (0.81–1.36) p=0.698 |
| Functional decline among survivors | 0.94 (0.80–1.10) p=0.446 | 0.95 (0.79–1.15) p=0.596 | 0.94 (0.80–1.11) p=0.473 | 0.94 (0.80–1.11) p=0.483 | 0.95 (0.79–1.15) p=0.619 |
| Death within 3 months | 0.68 (0.20–2.31) p=0.538 | 1.00 (0.27–3.73) p=0.999 | 0.74 (0.22–2.52) p=0.627 | 0.67 (0.20–2.27) p=0.519 | 0.98 (0.26–3.67) p=0.980 |

This table extends the original prespecified sensitivity analyses (Supplementary Table S7) to all eight outcomes. The primary analysis used a 72-h cutoff (N=423). The alternative definition used a 48-h cutoff (N=423). The landmark analysis was restricted to patients with an ICU LOS ≥3 days (N=420). The excluding never sitters analysis excluded the 19 patients who did not achieve sitting during the ICU stay (N=404). The ≤48 h + excluding never sitters analysis combined the 48-h cutoff with exclusion of never sitters (N=404). All models were estimated using overlap-weighted generalized linear models with a Poisson distribution, a log link, and robust variance estimators. Abbreviations: CI, confidence interval; ICU, intensive care unit; LOS, length of stay; RR, risk ratio.

**Supplementary Table S12**. **KCL cognitive domain: item-level analysis and alternative definitions.**

| **Cognitive measure** | **Delayed sitting (n=208)** | **Early sitting (n=149)** | **Adjusted comparison** | **P value** |
| --- | --- | --- | --- | --- |
| Q18: Memory (“Told by others of forgetfulness”) | 43/208 (20.7%) | 25/149 (16.8%) | RR 0.77 (0.46–1.29) | p=0.322 |
| Q19: Telephone use (“Able to look up phone number and call”) | 117/208 (56.2%) | 56/149 (37.6%) | RR 0.89 (0.67–1.17) | p=0.399 |
| Q20: Orientation (“Sometimes cannot remember today’s date”) | 54/208 (26.0%) | 41/148 (27.7%) | RR 0.97 (0.65–1.43) | p=0.864 |
| Cognitive domain impairment or death (primary composite definition) | 166/223 (74.4%) | 83/153 (54.2%) | RR 0.80 (0.67–0.97) | p=0.024 |
| Alternative: ≥2/3 items positive (more stringent) | 48/208 (23.1%) | 33/149 (22.1%) | RR 1.07 (0.67–1.70) | p=0.780 |
| KCL cognitive sum (0–3) | 1.03±0.83 | 0.82±0.93 | Mean diff: −0.21 | p=0.028 |
| KCL total score (0–25) | 10.98±5.52 | 10.89±7.05 | Mean diff: −0.09 | p=0.897 |

The Kihon Checklist (KCL) cognitive domain consists of three items (Q18–Q20). The primary definition of cognitive impairment used ≥1 of 3 items positive (per KCL scoring convention). Adjusted RR (95% CI) obtained using overlap-weighted Poisson regression. Q18 assesses memory, Q19 assesses telephone use (an instrumental activity of daily living item), and Q20 assesses orientation to date. Item-level analyses included survivors with available responses to each item (delayed n=208, early n=149; n=148 for Q20).

**Supplementary Table S13. Effective sample size after overlap weighting.**

| **Analysis** | **Original sample size** | **Effective sample size** |
| --- | --- | --- |
| Primary analysis (≤72 h) | 423 | 298.8 |
| Alternative definition (≤48 h) | 423 | 250.1 |

Effective sample size (ESS) was calculated as (∑w)^2^/∑w^2^ to quantify the amount of statistical information retained after overlap weighting. ESS values closer to the original sample size indicate minimal loss of precision due to weighting.

**Abbreviations:** ESS, effective sample size.

**Supplementary Table S14. Sensitivity analysis for unmeasured confounding using E-values.**

| **Outcome** | **Adjusted RR** | **95% CI** | **E-value (Point estimate)** | **E-value (CI limit)** |
| --- | --- | --- | --- | --- |
| Cognitive domain impairment or death | 0.80 | 0.67–0.97 | 1.80 | 1.21 |
| Readmission or death | 0.53 | 0.32–0.88 | 3.15 | 1.52 |

E-values represent the minimum strength of association that an unmeasured confounder would need to have with both early sitting and the outcome, on the risk ratio scale, to fully explain away the observed association, conditional on the measured covariates. E-values were calculated for both the point estimate and the confidence interval limit closest to the null value.

Abbreviations: RR, risk ratio; CI, confidence interval.
